# Probing Local Structural Variations in Metal–Organic Framework Thin Films Using Nano-FTIR

**DOI:** 10.1021/acs.langmuir.6c02252

**Published:** 2026-05-23

**Authors:** Yukihiro Matsumoto, Kento Takenaka, Hiromasa Sato, Yuto Fujita, Toshiki Sugimoto, Tomoko K. Shimizu

**Affiliations:** † Department of Applied Physics and Physico-Informatics, Faculty of Science and Technology, Keio University, 3-14-1 Hiyoshi, Kohoku-ku, Yokohama, Kanagawa 223-8522, Japan; ‡ Department of Materials Molecular Science, 88301 Institute for Molecular Science, Okazaki, Aichi 444-8585, Japan; § Graduate Institute for Advanced Studies (SOKENDAI), Okazaki, Aichi 444-8585, Japan

## Abstract

Precise control over coordination structures and orientation is crucial for integrating metal–organic framework (MOF) thin films into functional devices. In this study, we combined atomic force microscopy with nano-Fourier transform infrared spectroscopy to definitively characterize local coordination bonding in a representative MOF thin film, NAFS-1, fabricated at the air/liquid interface. Our nanoscale analysis revealed structural inhomogeneities often obscured in macroscopic measurements. Additionally, we identified several characteristic regions in NAFS-1, including areas with uncoordinated carboxyl groups and with large, elevated features exhibiting effective coordination. Furthermore, omitting the water-rinsing step, usually conducted after transferring a film to the substrate to eliminate surface contaminants, increased structural inhomogeneity while promoting coordination bonding on average. These results highlight the importance of local characterization in understanding film crystallinity and uniformity and provide critical insights into the MOF thin-film formation mechanism at the air/liquid interface.

## Graphical abstract

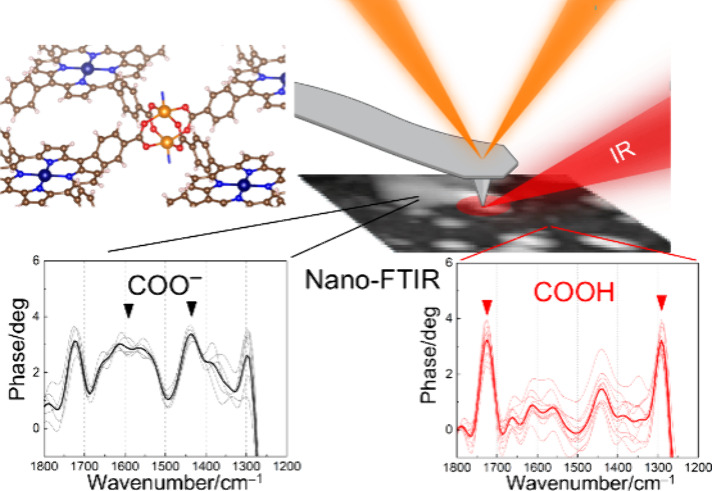

## Introduction

Metal–organic frameworks (MOFs) are porous crystalline materials formed through coordination between metal ions and organic ligands, leading to the formation of periodically arranged nanoscale pore networks. While traditional MOFs are typically in powder form, two-dimensional (2D) MOFs consist of atomically thin sheets or layered structures, resulting in a high surface area and distinct chemical and in-plane electronic properties.^1–3^ Development of 2D MOFs has expanded the application scope of MOFs beyond gas storage and catalysis to include sensors, separation filters, and electronic devices.^4–11^ Recent studies have demonstrated improved control over the film uniformity and continuity of 2D MOFs;^12,13^ however, significant challenges remain in precisely controlling properties such as thickness, crystallinity, and layer orientation.^14^ Achieving such a control is essential for tailoring 2D MOF properties for specific applications.

A key factor governing the structural integrity and stability of 2D MOFs is the coordination bonding between metal ions and polar functional groups in organic ligands. Structural defects, such as missing metal ions or unbound functional groups leading to incomplete coordination, can markedly impact the pore architecture and consequently alter the material properties. Therefore, techniques capable of probing local bonding states and defect distributions are crucial for understanding and ultimately controlling the structural irregularities in 2D MOFs.

2D MOFs have been analyzed using various techniques, including grazing-incident X-ray diffraction (GIXRD),^3,14,15^ infrared (IR) spectroscopy,^14–17^ and ultraviolet–visible (UV–vis) absorption spectroscopy.^15,17,18^ Nevertheless, the spatial resolution of these conventional methods is fundamentally limited by the probe spot size, hindering direct evaluation of local structures at the nanoscale. Meanwhile, scanning probe microscopy allows for local structural characterization. Atomic force microscopy (AFM) has been widely employed to investigate the nanoscale morphology of organic porous thin films;^19–22^ however, it provides only limited insight into coordination states, necessitating techniques capable of probing chemical bonding. Scanning tunneling microscopy (STM) operated under ultrahigh vacuum (UHV) conditions can resolve structures at even a higher spatial resolution than AFM operated in ambient conditions and can additionally provide information regarding local electronic structures. However, most STM studies have focused on monolayer MOFs prepared by evaporating molecular powders under vacuum.^23–26^ Although UHV-prepared 2D MOFs serve as valuable model systems, their structures may substantially differ from solution-processed 2D MOFs because of distinct substrate interactions and growth conditions.^27,28^ Therefore, establishing structural characterization methods suitable for solution-processed 2D MOFs is essential to advance their practical applications.

IR scattering-type scanning near-field optical microscopy (IR *s*-SNOM), in conjunction with nano-Fourier transform IR spectroscopy (nano-FTIR), facilitates probing of local vibrational states, enabling chemical analysis of coordination bonding involving polar functional groups under ambient conditions.^29^ Nano-FTIR has been utilized across various materials and systems, encompassing soft matter,^30–34^ nano- and molecular structures,^35–39^ functional thin films,^40–42^ interfaces,^43–45^ and extraterrestrial samples.^46,47^ Studies have applied nano-FTIR to MOF crystals and films, demonstrating its capability to reveal coordination states,^48,49^ host–guest interactions,^50^ and crystallization processes.^51^ Despite these advancements, characterization of 2D MOFs using this technique is not yet well established. While similar methods, such as photothermal AFM–nano-IR and photoinduced force microscopy, have been used to investigate initial stages of film growth in a SURMOF (surface-mounted MOF),^52^ evaluation of local bonding states and film orientation remains largely unexplored.

In this study, we present a nanoscale structural analysis of NAFS-1 (nanofilm of metal–organic frameworks on surfaces no. 1),^15^ a representative 2D MOF, using nano-FTIR to identify the local variations in its structure and coordination states. NAFS-1 was fabricated at the air/liquid interface using a Langmuir trough and subsequently transferred onto solid substrates.^8,15^ This fabrication method was chosen for its simplicity and good control over the film thickness. We successfully resolved local structural inhomogeneities otherwise obscured in macroscopic measurements. Our analysis revealed several characteristic regions in NAFS-1, including areas with significant amounts of uncoordinated carboxyl groups, particle-like features, and large domains exhibiting a higher degree of coordination. Furthermore, we found that omitting the water-rinsing step increased structural inhomogeneity at the nanoscale, as observed by AFM; yet, this omission promoted coordination bonding on average—a finding corroborated by the macroscopic FTIR and UV–vis absorption spectra. These results highlight the necessity of local structural and chemical characterization in evaluating 2D MOFs, providing critical insights for their formation processes.

## Experimental Section

### Substrate Preparation

Si substrates (1 cm × 1 cm) were prepared by cutting a Si wafer using a diamond cutter. The substrates were cleaned via sequential sonication in chloroform, acetone, and ethanol for 30 min each.

### Solution Preparation

5,10,15,20-Tetrakis­(4-carboxyphenyl)­porphyrinato-cobalt­(II) (CoTCPP, 1.9 mg, Porphyrin Systems, 98%; Figure 1a) and pyridine (py, 4 μL, Junsei Chemical, ≥99.5%; Figure 1b) were dissolved in a 10 mL methanol (Kanto Chemical, ≥99.8%)/chloroform (Fujifilm Wako Pure Chemical, ≥99.8%) mixture (1:3 v/v) and sonicated for 10 min. The subphase, a 0.1 mol/L copper chloride solution, was prepared by dissolving CuCl_2_/2H_2_O powder (Kanto Chemical, ≥99.9%) in Milli-Q water and stirring with a glass rod.

**Figure 1:**
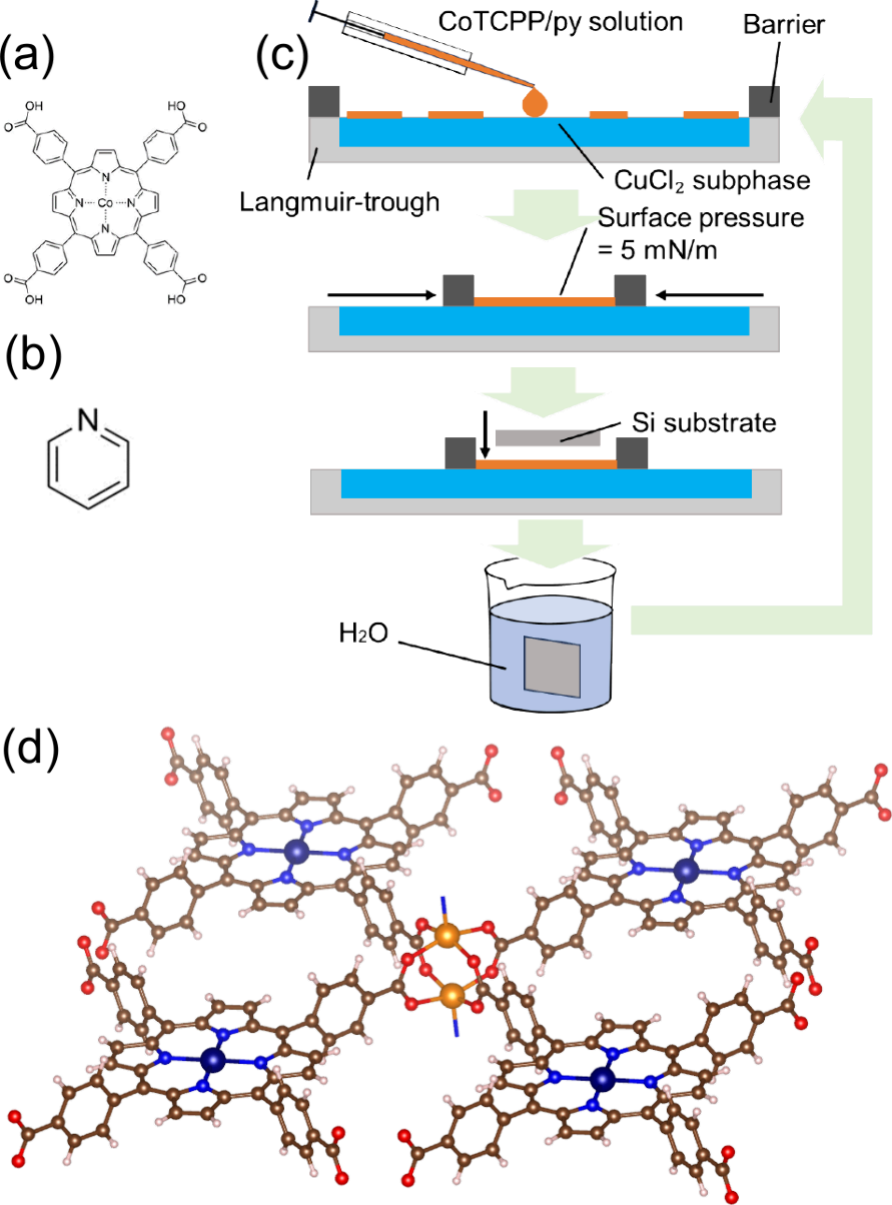
(a) Chemical structures of (a) CoTCPP and (b) pyridine. (c) Schematic of the film fabrication process using a Langmuir trough. (d) Structural model of NAFS-1, generated by VESTA using the crystallographic information file given in ref 18. Pyridine molecules are omitted to emphasize the central paddle-wheel structures shown. Color code: Cu, orange; Co, dark blue; C, brown; N, blue; O, red; and H, white.

### Film Fabrication

The fabrication process is illustrated in Figure 1c. Initially, a KSV NIMA Langmuir mini-trough (195 × 50 × 4 mm^3^, 57 L) was filled with a CuCl_2_ solution, which served as the subphase. Subsequently, the CoTCPP/py solution (33.3 μL) was delicately spread onto the subphase using a microsyringe. The resulting thin film, as depicted in the schematic model in Figure 1d, was compressed toward the center of the trough by two Teflon barriers at a constant speed of 10 mm/min until the surface pressure (π) reached 5 mN/m. Throughout compression, the π-mean molecular area (*A*) isotherm was monitored using a Pt Wilhelmy plate. The MOF thin film was then transferred onto a substrate using the horizontal dipping method, followed by rinsing in Milli-Q water for about 3 min. This process was repeated for 10 cycles unless otherwise noted. This sample is referred to as “NAFS-1”. For comparison, another sample was prepared without the final water-rinsing steps, which is referred to as “NAFS-1 without water rinsing”.

### Control Samples

For comparison, we prepared control samples by using the constituent materials of NAFS-1. These samples were prepared by (1) spreading a CoTCPP solution on a water subphase and subsequently transferring the floating material onto a Si substrate (repeated ten times (CoTCPP/water)) and (2) making a Si substrate briefly touch the surface of a CuCl_2_ solution (repeated ten times (CuCl_2_)). These samples did not undergo a water rinsing.

### AFM and Nano-FTIR

AFM and nano-FTIR analyses were conducted on multilayered samples using a commercial IR *s*-SNOM system (neaSNOM, Neaspec GmbH). For monolayer samples, a custom-built IR *s*-SNOM system (based on neaSNOM, Neaspec GmbH) equipped with a high-repetition-rate pulsed IR laser was utilized (refer to the Supporting Information for specifics). PtIr-coated AFM tips (Arrow NCPt, NanoWorld; resonant frequency: 285 Hz, spring constant: 42 N/m) and their gold-coated tips were used for data acquisition on multilayer and monolayer samples, respectively. AFM was operated in the tapping mode with an oscillation amplitude of approximately 100 nm. During interferogram collection, the reference mirror of the asymmetric Michelson interferometer was moved at a speed of 7.3 μm/s across a 150 μm range, corresponding to a frequency resolution of 33 cm^–1^. The interferogram was recorded at 1024 pixels, with an acquisition time of 20 ms per pixel. Before measurements, an approach curve was obtained on a Si substrate, plotting the near-field scattering intensities against the tip–sample distance over 200 nm with 200 pixels, at a speed of 50 ms/pix. Based on this approach curve (Supporting Information Figure S1), the third-harmonic signal provided adequate localization and a reasonable signal-to-noise ratio; thus, it was used for subsequent analysis.

Measurements were conducted at multiple locations on samples and bare Si substrates. The amplitude and phase spectra of the scattering coefficients, defined as σ­(ω) = *s*(ω)*e* ^*i*ϕ(ω)^, were acquired through Fourier transformation of the interferogram from the asymmetric Michelson interferometer. The phase spectrum ϕ­(ω), approximating the imaginary part of the dielectric function of the sample, served as the nano-FTIR spectrum. For each phase spectrum of the sample (ϕ_SAMPLEonREF_), the reference spectra were recorded at three distinct locations on the bare substrate (ϕ_REF1_, ϕ_REF2_, ϕ_REF3_), which were then subtracted to generate the differential spectra: ϕ_SAMPLE_^*N*^ = ϕ_SAMPLEonREF_ – ϕ_REFN_ (*N* = 1,2,3). The ultimate nano-FTIR spectrum of the sample under the probe (AFM tip) was defined as the average of the three differential spectra: ϕ_SAMPLE_ = (ϕ_SAMPLE_^1^ + ϕ_SAMPLE_^2^ + ϕ_SAMPLE_^3^)/3. Subsequently, a linear baseline correction was applied. The experimental noise level was evaluated by measuring the nano-FTIR spectra of a Si substrate referenced to its own spectrum (Figure S2). Based on this result, we defined a phase shift of 1° as the threshold for signal identification throughout the study of the multilayered samples.

### Macroscopic IR Spectroscopy

The spectra of NAFS-1 transferred 30 times onto Si substrates with and without water rinsing were recorded in the transmission mode using JASCO FT/IR-6600. A clean Si substrate served as the reference. Data were collected at a resolution of 4 cm^–1^, averaging 1024 scans per spectrum. All measurements were performed at room temperature under a nitrogen atmosphere.

### UV–Vis Absorption Spectroscopy

The UV–vis absorption spectra of NAFS-1 on Si substrates, both with and without water rinsing, were obtained via the diffuse-reflectance method using a spectrophotometer with an integrating sphere (JASCO V-670) at room temperature.

## Results and Discussion

An AFM topographic image of NAFS-1 on a Si substrate reveals small islands across the surface (Figure S3a). Enlarged images show that these lower-lying islands possess lateral dimensions of tens to hundreds of nanometers (Figure 2a–c), which were defined as “domains” in a previous study.^15^ Typical heights of these islands are in the range of 10–20 nm (Figure S3b,c). Several prominent islands with heights of approximately 50 nm are also observed, including small aggregates of several particles (Figure 2b and Figure S3b,c) and larger, flatter features (Figure 2a,c and Figure S3d,e).

**Figure 2:**
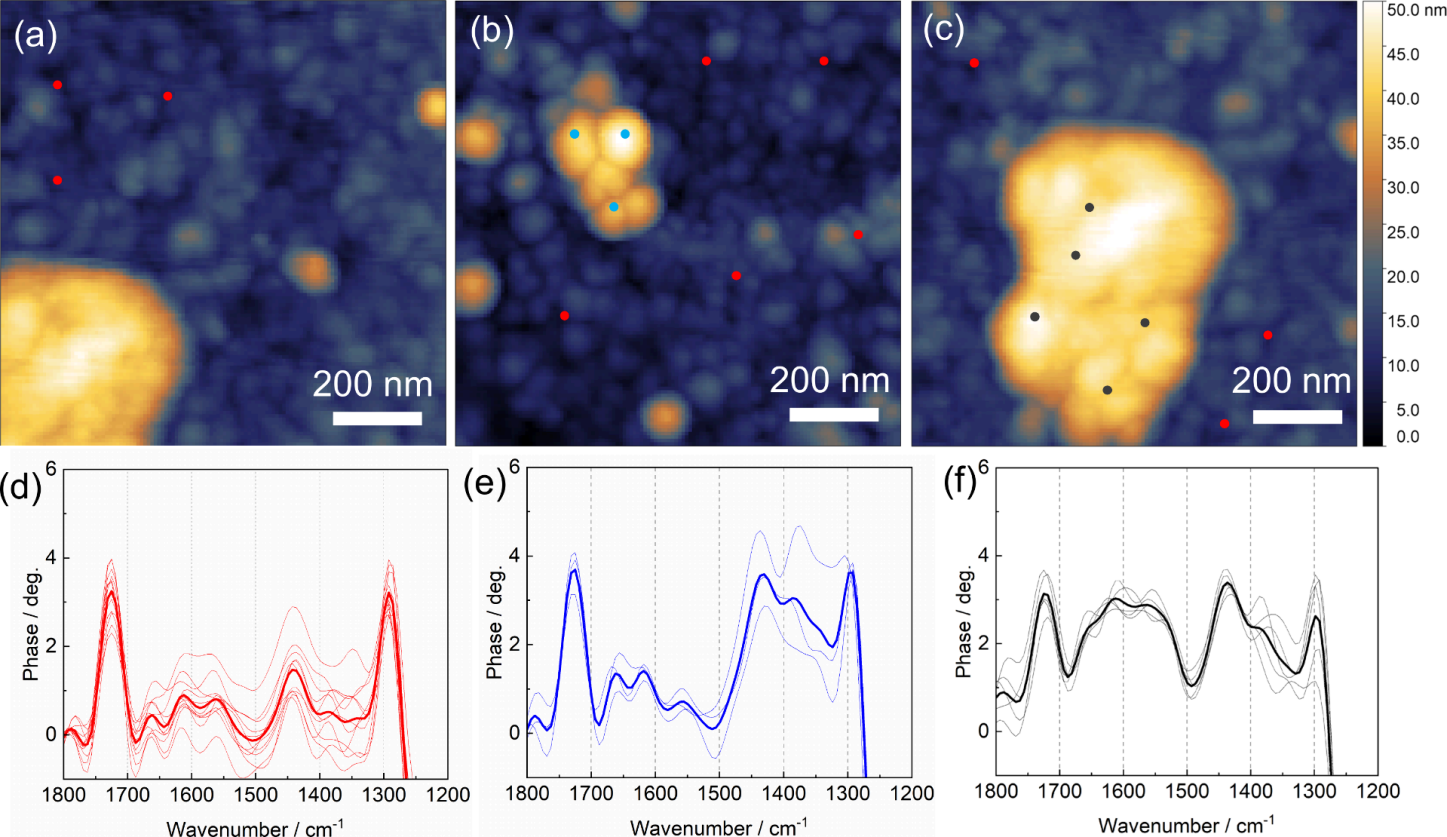
(a–c) AFM topography of a 10-layer NAFS-1 sample prepared by the conventional method (with water-rinsing steps after each cycle). The size of dots corresponds to φ ∼20 nm, an approximate size of near-field light confinement. (d–f) Nano-FTIR spectra obtained from locations indicated by corresponding colors in (a–c). Thin lines represent individual spectra from 11 red spots (3 in (a), 5 in (b), and 3 in (c)), 3 blue spots in (b), and 5 black spots in (c). Thick lines indicate their respective averages.

The nano-FTIR spectra of the lower regions (indicated by red spots in Figure 2a–c) exhibit two prominent peaks: one at 1720 cm^–1^, attributed to the C=O stretching mode of carboxylic acid (COOH) groups, and another at 1290 cm^–1^, which is likely associated with the C–OH stretching mode and formate bending mode, although the latter assignment has not been explicitly reported in previous studies.^8,15^ These peaks are dominant and consistently observed across different sample batches, despite minor spectral variations (Figure S4 in the Supporting Information). While weaker peaks are occasionally observed at 1430 cm^–1^, assigned to the symmetric stretching vibration of COO^–^, and at 1610 cm^–1^, corresponding to the antisymmetric stretching vibration of COO^–^, their intensities remain low relative to those of the COOH peaks. These observations indicate that while the formation of coordination bonds with Cu ions is inferred, complete coordination is not achieved throughout the lower regions of the film; instead, a significant fraction of noncoordinated COOH groups persists within the film.

In addition to these lower regions, the NAFS-1 samples contain localized particle-like features (Figure 2b) and larger islands (Figure 2a,c), both with heights of approximately 40–50 nm (Figure S3b–e). These heights significantly exceed the expected thickness of the 10-layer NAFS-1 (∼12 nm). The nano-FTIR spectra of small particles (blue spots in Figure 2b) exhibit three peaks at 1290, 1430, and 1720 cm^–1^, similar to those of the lower regions; however, the relative intensity of the 1430 cm^–1^ peak is substantially higher (Figure 2e). While this suggests more efficient coordination bonding, the absence of the peak at 1610 cm^–1^ implies that the expected MOF orientation is not achieved. Given that nano-FTIR is capable of detecting vibrational modes with dipole components oriented perpendicular to the substrate (see the Supporting Information Figure S5 for the relationship between each vibrational mode and its net dipole),^53^ the peak at 1610 cm^–1^ is expected to appear if the films are horizontally aligned. Based on these observations, we conclude that these particles represent 3D MOF crystallites rather than a continuous, uniform film. Furthermore, the larger islands (Figure 2c) exhibit a more complex spectral profile with four distinct peaks (Figure 2f). A notable increase in the intensity is observed in the 1550–1650 cm^–1^ region, suggesting not only more effective coordination but also a greater diversity in the coordination environment and orientation. In particular, the broadening of the peaks in this range may reflect the variations in the coordination number or local structural distortions within the so-called paddle-wheel units.

To confirm if the spectral features observed for NAFS-1 originate from simple residues of the raw materials, we prepared two additional control samples. The AFM images of the CoTCPP/water sample (Figure 3a,b) reveal smaller domains than those observed for NAFS-1. The average spectrum shown in Figure 3c exhibits two main peaks at 1720 and 1290 cm^–1^, indicative of COOH groups. Furthermore, the intensities at 1430 and 1610 cm^–1^ remain below the noise level, although slight increases are observed around those regions. These minor features cannot be confidently assigned as peaks, as the signal-to-noise ratio is limited by our noise level of approximately 1° (Figure S2). The AFM images of the CuCl_2_ sample reveal round particles (Figure 3d,e), which resemble particle-like features observed in the NAFS-1 sample (Figure 2b). However, the average nano-FTIR spectrum of CuCl_2_ shows no distinct peaks (Figure 3f), thereby ruling out the possibility that the features found in the NAFS-1 sample are simply residual CuCl_2_.

**Figure 3:**
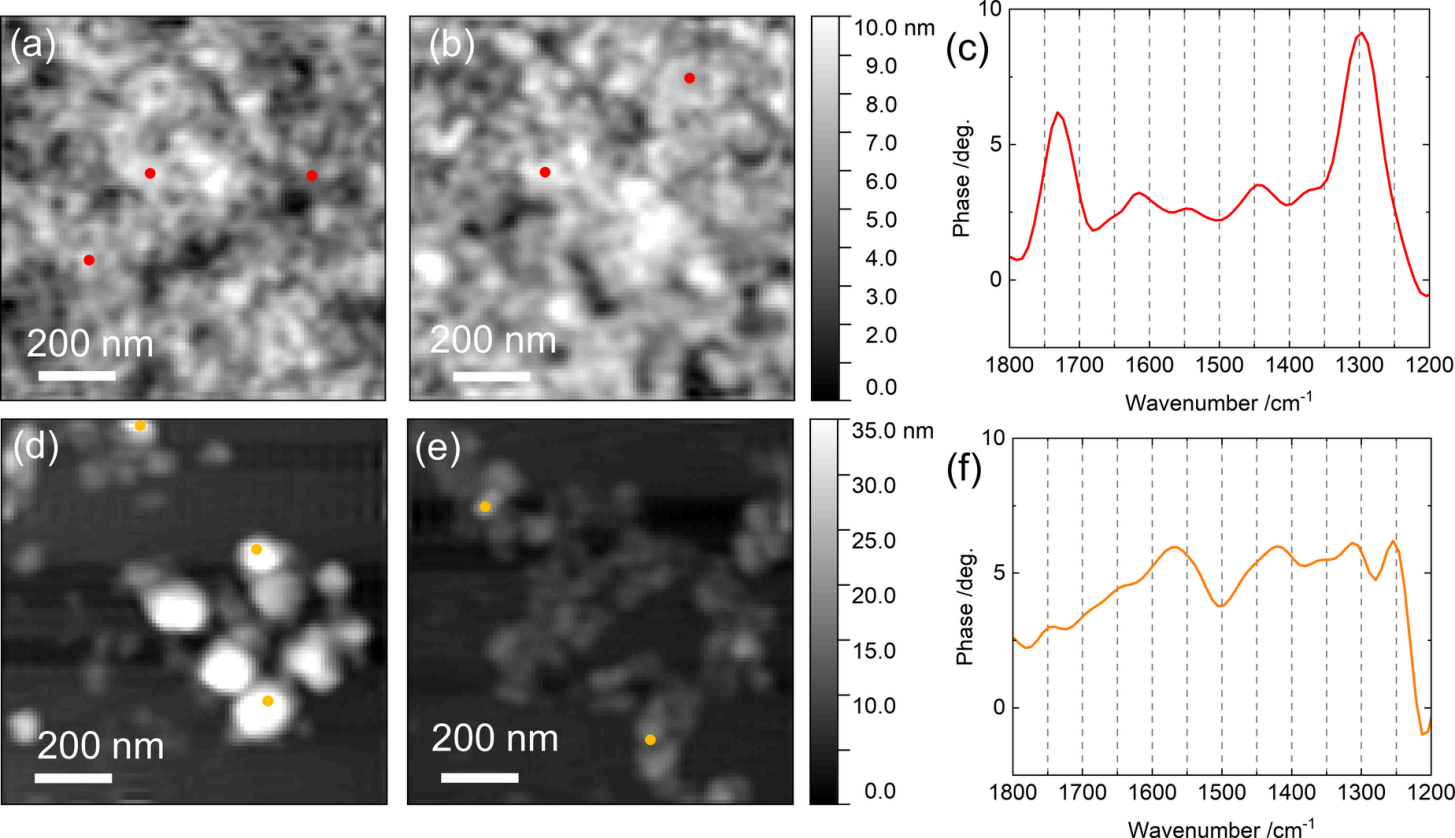
(a, b) AFM topography of a CoTCPP sample prepared by 10 transfer cycles from a water subphase without water-rinsing steps after each cycle (CoTCPP/water). No copper ions or pyridine were used during fabrication. (c) Averaged nano-FTIR spectrum from five locations depicted in (a) and (b). (d, e) AFM topography of a Si substrate subjected to 10 cycles of contact with a CuCl_2_ solution without water rinsing after each cycle (CuCl_2_). (f) Averaged nano-FTIR spectrum from five locations illustrated in (d) and (e).

To clarify the origin of the noncoordinated COOH groups in the NAFS-1 samples, we further examined NAFS-1 prepared by just one transfer, having a thickness of ∼1.2 nm, consistent with the reported monolayer thickness^15^ (Figure S6). To overcome technical challenges because of the extremely low intensities of the nano-FTIR signals, measurements were conducted using a custom-built nano-FTIR system purged with nitrogen and equipped with a high-repetition-rate pulsed IR laser (see the Supporting Information for details). The spectrum of the domain interiors reveals a dominant peak at 1720 cm^–1^ and a negligible signal at 1610 cm^–1^ (Figure S6c). This result indicates that incomplete coordination is inherent to the entire area of the monolayer NAFS-1, rather than being localized at domain edges, and is not an artifact of the multilayer stacked structure.

The results obtained so far indicate that both monolayer and multilayered NAFS-1 contain a substantial number of uncoordinated carboxy groups distributed throughout the film, regardless of their location within or at the periphery of the domain. More effective coordination bonding is achieved only in certain irregular high-profile features, such as aggregated particles and large islands much higher than the expected 10-layer thickness. Because these features have not been reported in previous studies and are only observed in our experiments when the AFM scan range is sufficiently large (e.g., 5 μm), these features can be attributed to secondary 3D growth at the air/liquid interface. In other words, rather than simple impurities, they are localized 3D agglomerates whose growth deviates from the ideal 2D growth. Although these nonideal 3D features are expected to be removed during the water-rinsing steps after transferring to the substrate, they can occasionally persist.

To investigate the effect of water rinsing more thoroughly, we prepared samples by omitting the water-rinsing step. The large-scale AFM image reveals a highly inhomogeneous surface, characterized by numerous large islands and particles distributed across the entire area (Figure S7a). The enlarged images of areas containing these elevated features (Figure 4a and Figure S7b–e) also reveal small islands (red spots).

**Figure 4:**
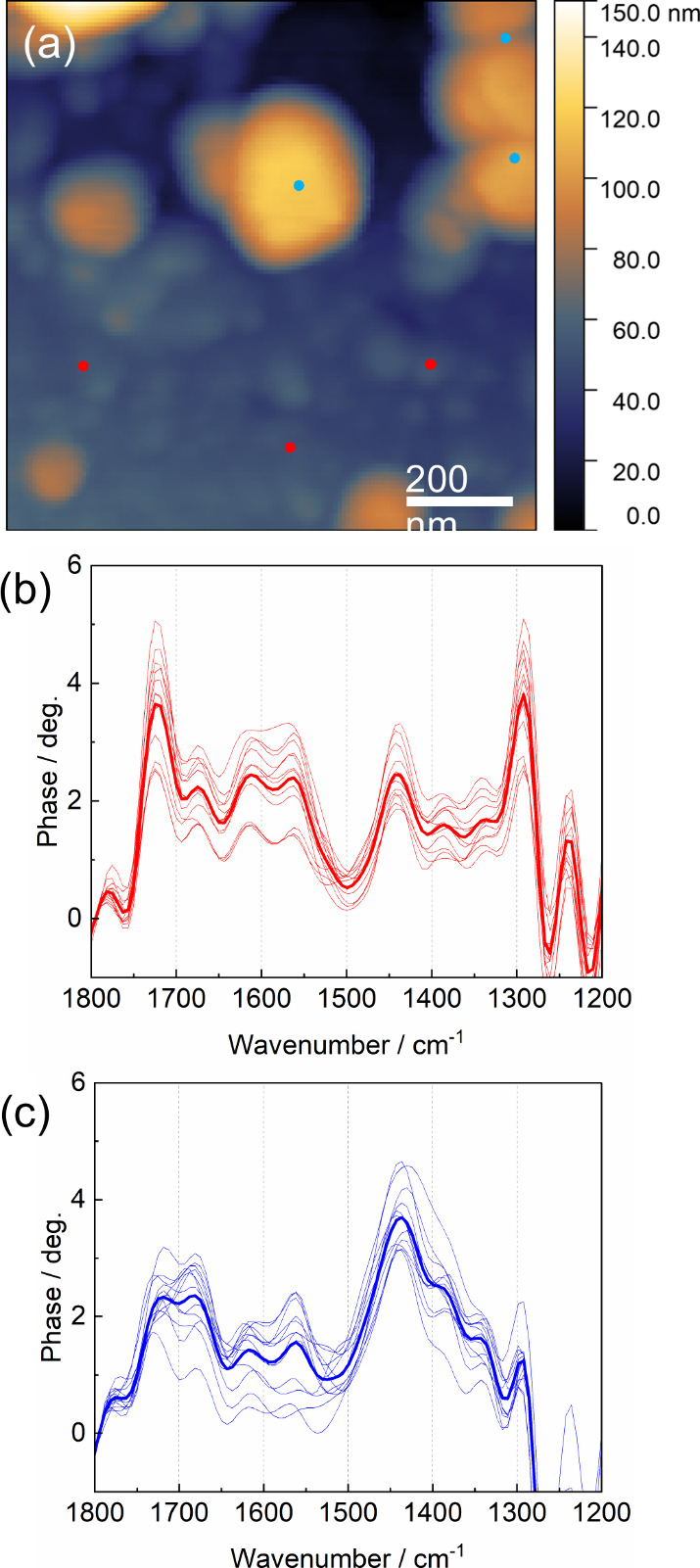
(a) AFM topography image of a 10-layer NAFS-1 sample prepared without water-rinsing steps after each cycle. (b, c) Nano-FTIR spectra obtained from 12 red and 12 blue spots, respectively. These include the representative locations indicated in (a) and additional locations shown in Figure S7. Thin lines represent individual spectra, and thick lines indicate their respective averages.

The nano-FTIR spectra of the lower areas (red spots) exhibit four peaks, similar to those displayed by the rinsed sample, albeit with variations in the intensity (Figure 4b). The two COOH-related peaks indicate the presence of noncoordinating bonds within these regions. By contrast, the spectra of the larger, high features (blue spots) show an intense peak at 1430 cm^–1^, suggesting extensive MOF coordination (Figure 4c). However, the crystallinity and bond orientations seem to differ from those observed for the particles and high features of the rinsed NAFS-1 (Figure 2b,c,e,f). We attribute these differences to the variations in the crystallinity and bonding orientation rather than the effect of water rinsing on the formation mechanism. Instead, various 3D crystallites are likely formed at the air/liquid interface during fabrication, which are simply removed in the subsequent water-rinsing step.

Finally, to evaluate how this structural diversity is reflected in the bulk average, we compared our nanoscale observations with the conventional macroscopic data. Overall, the macroscopic transmission-mode FTIR spectra (Figure S8) are in good agreement with the nano-FTIR results; both exhibit two distinct COOH-related peaks at ∼1725 and 1275 cm^–1^, along with the broad features assigned to COO^–^ vibrational modes. A closer inspection of the macroscopic FTIR spectra reveals subtle differences between NAFS-1 with and without water rinsing. While the nonrinsed NAFS-1 exhibits peaks around 1600 and 1440 cm^–1^, these COO^–^-related peaks are less apparent in the rinsed NAFS-1, superficially suggesting more effective coordination in the nonrinsed sample. However, given that transmission-mode FTIR primarily detects dipole components parallel to the substrate, the appearance of the 1600 cm^–1^ peak in the nonrinsed sample indicates the presence of nonhorizontally aligned components. As revealed by our nano-FTIR observations, these nonhorizontal components are likely associated with 3D particles and elevated features.

The trend of the macroscopic “improvement” in coordination in the nonrinsed NAFS-1 is also evident in the UV–vis absorption data (Figure S9), where the nonrinsed sample exhibits a clear redshift. This shift implies the expansion of the π-conjugated system through more extensive coordination;^8^ however, our nano-FTIR results indicate that this is an artifact driven by the presence of 3D microcrystals and large islands in the nonrinsed samples. The removal of these 3D features, which likely lack the specific face-to-face stacking proposed in the NAFS-1 model,^15^ results in the dominance of 2D stacking regions. This structural transition may account for the observed blue shift upon rinsing,^54^ although other factors arising from structural complexity may also contribute to these spectral variations.

## Conclusions

We employed AFM combined with nano-FTIR to characterize 10-layer NAFS-1 samples fabricated at the air/liquid interface. The variations in the structural features and nano-FTIR spectra of the samples revealed inhomogeneities in coordination bonding and orientation. In NAFS-1 prepared by the conventional method, which incorporated the water-rinsing step after each film transferring cycle, uncoordinated carboxy groups were found to be distributed throughout the film. Certain sparse features, such as particle-like aggregates and large, elevated features, exhibited enhanced coordination, as evidenced by the increased intensity of the carboxylate (COO^–^) vibrational peaks. The AFM images and nano-FTIR spectra exhibited more pronounced structural inhomogeneities when the post-transfer water-rinsing step was omitted; however, macroscopic spectroscopies indicated more effective coordination and expanded conjugation. Our findings emphasize the necessity of integrating nanoscale imaging with spatially resolved spectroscopy to identify the local crystallinity and orientation of MOF thin films. This approach provides a crucial framework for evaluating and optimizing the structural integrity of this class of materials, preventing misinterpretation of macroscopic data that will otherwise obscure irregularities because of the averaging effects.

While this study focused on NAFS-1 as a well-established model system to demonstrate the capabilities of our nanoscale approach, it is important to note the recent extensive advancements in 2D MOFs fabricated at air/liquid interfaces and via other sophisticated techniques. Future research should extend this integrated AFM and nano-FTIR methodology to a broader range of 2D MOFs with higher crystallinity and superior orientation. Furthermore, optimizing substrate choice and refining the understanding of signal emergence will be essential for a more definitive determination of the film orientation. Such investigations will further define the scope and robustness of this methodology while providing useful insights into the underlying film-growth mechanism.

## Supplementary Material



## Notes

Approach curves; nano-FTIR spectrum of a Si substrate referenced to itself; large-scale AFM image of NAFS-1, height profiles; AFM images of other NAFS-1 samples; structural models of COO^–^ symmetric and antisymmetric stretching vibrations; AFM image, height profile, and nano-FTIR spectra of monolayer NAFS-1 on Si with detail of the custom-made IR *s*-SNOM system used for monolayer measurements; large-scale and enlarged AFM images of 10-layer NAFS-1 prepared without water rinsing, height profiles; and macroscopic transmission-mode FTIR and UV–vis absorption spectra of NAFS-1 and NAFS-1 prepared without water rinsing (PDF)
