# Supplementary material for: Probing Local Structural Variations in Metal–Organic Framework Thin Films Using Nano-FTIR
Source: Langmuir. 2026 May 23;42(22):16052–9. doi: 10.1021/acs.langmuir.6c02252 (PMC13262026; doi:10.1021/acs.langmuir.6c02252)
Supplement: Supplementary file 1 [file la6c02252_si_001.pdf]

Supporting Information

Probing Local Structural Variations in Metal–Organic Framework Thin Films Using Nano-FTIR

Yukihiro Matsumoto[1+], *Kento Takenaka*[1+], *Hiromasa Sato*[2], *Yuto Fujita*[1], *Toshiki Sugimoto*[2,3], *Tomoko K. Shimizu*[1*]

1. Department of Applied Physics and Physico-Informatics, Faculty of Science and Technology, Keio University, 3-14-1 Hiyoshi, Kohoku-ku, Yokohama, Kanagawa 223-8522, Japan.

2. Department of Materials Molecular Science, Institute for Molecular Science, Okazaki, Aichi 444-8585, Japan.

3. Graduate Institute for Advanced Studies (SOKENDAI), Okazaki, Aichi 444-8585, Japan.

+These authors contributed equally.

Corresponding Author:
**T. K. Shimizu:* tshimizu@appi.keio.ac.jp

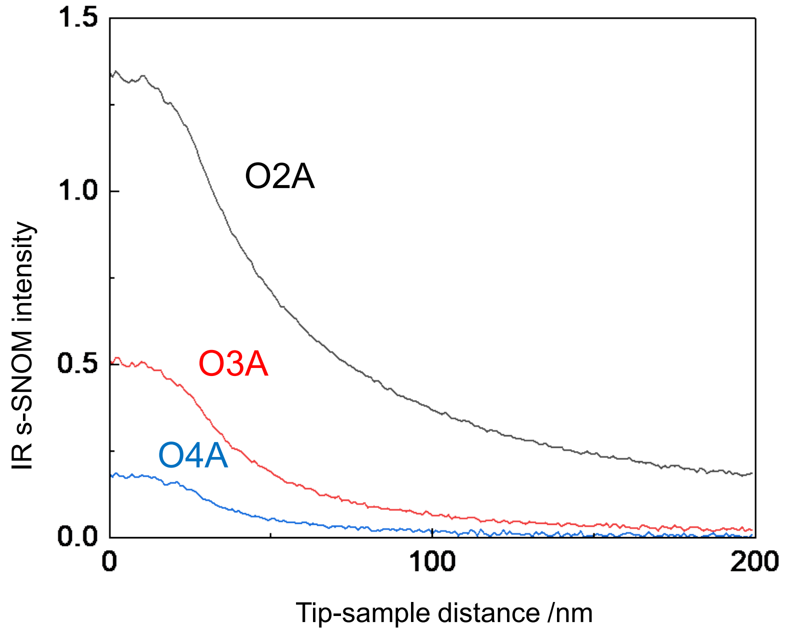

Figure S1. Approach curves displaying the intensity at frequencies corresponding to the second (black), third (red), and fourth (blue) harmonics of cantilever oscillation as a function of tip-sample distance, on a Si substrate. The curve was obtained over a 200 nm range with 200 pixels at a rate of 50 ms per pixel.

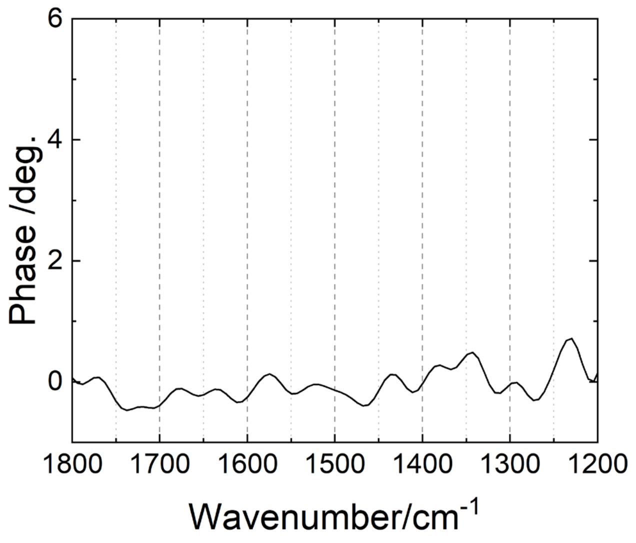

Figure S2. Nano-FTIR spectrum of a Si substrate referenced to itself to assess the experimental noise level. Only phase peaks exceeding 1° are considered meaningful throughout this study.

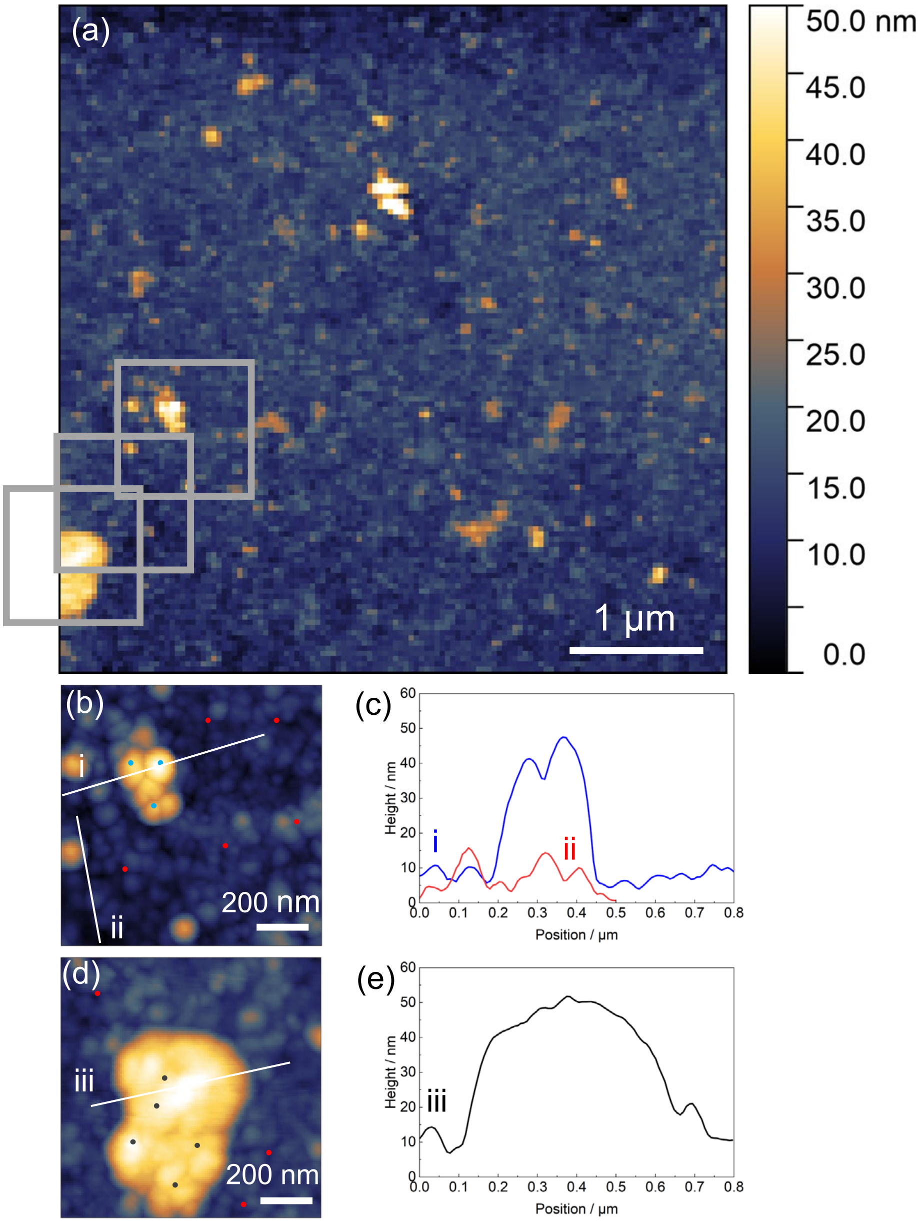

Figure S3. (a) Large-scale AFM topography of 10-layer NAFS-1 prepared in the conventional method with water-rinsing steps. (b,d) Enlarged AFM images, which are identical to those in Figures 2(b) and 2(c). (c,e) Height profiles along the lines indicated in (b) and (d), respectively.

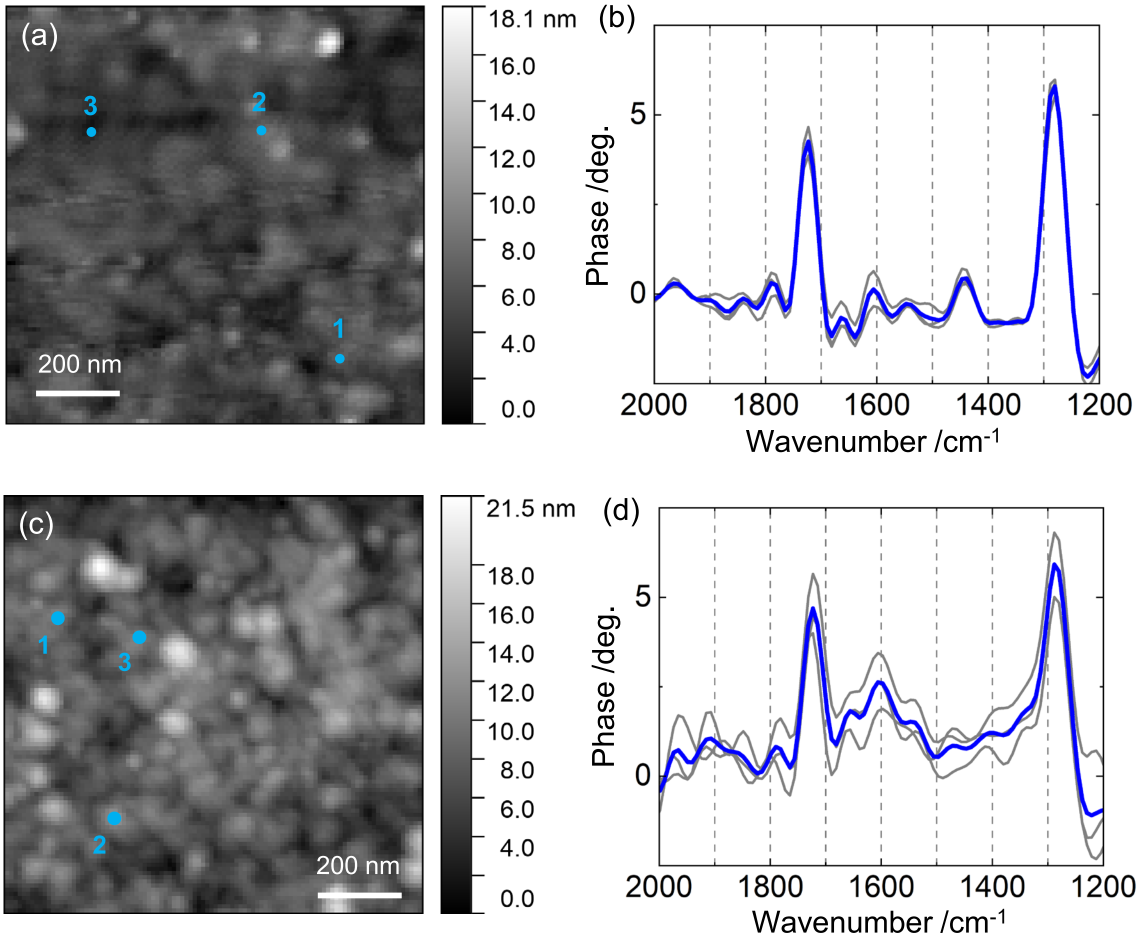

Figure S4. AFM images and nano-FTIR spectra from three locations (gray) and respective average curves (blue) of two 10-layer NAFS-1 samples prepared in the same way as that in Figure 2.

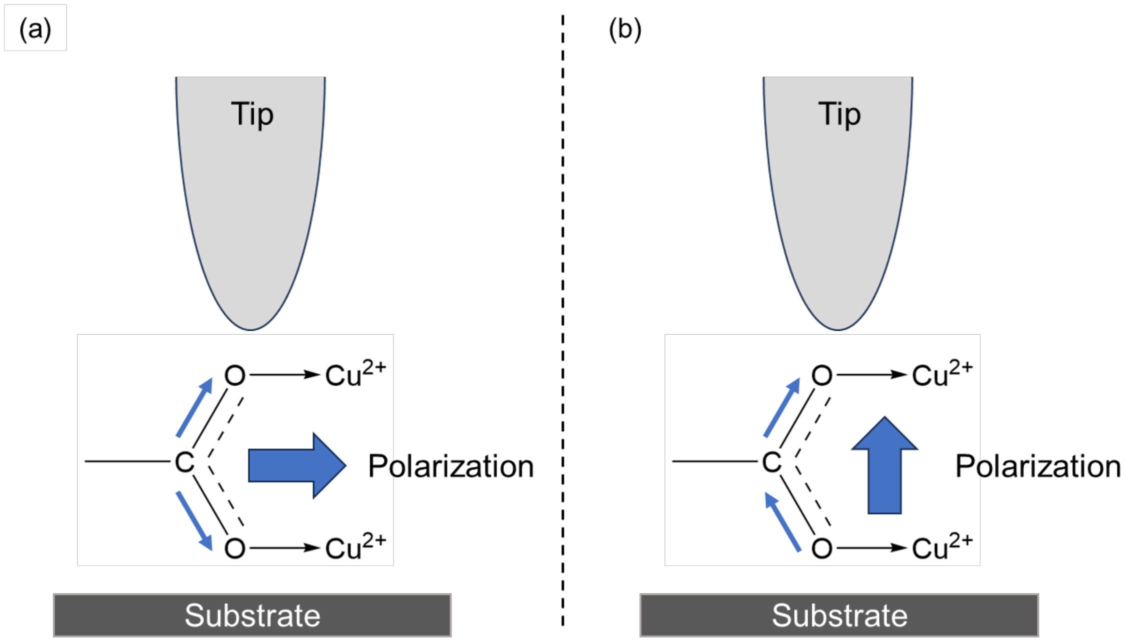

Figure S5. Structural models of (a) COO^- symmetric and (b) COO^- antisymmetric stretching vibrations. The symmetric and antisymmetric modes exhibit dipole components parallel and perpendicular to the substrate, respectively.

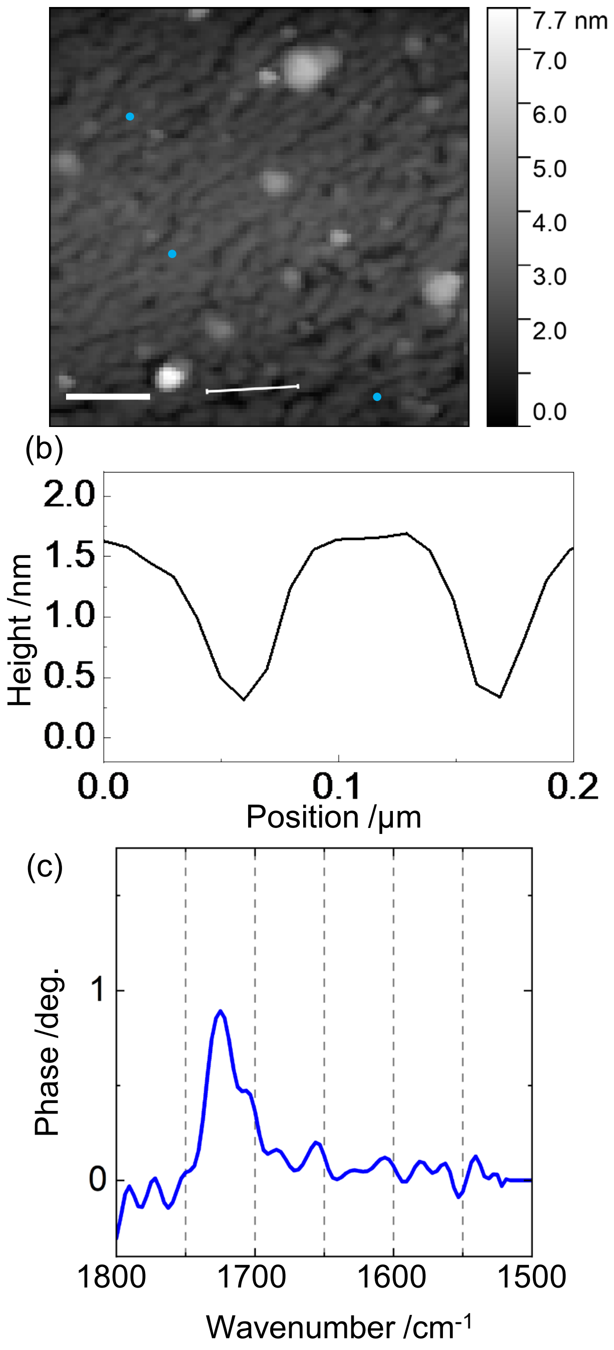

Figure S6. (a) AFM topography of NAFS-1 transferred once onto a Si substrate. (b) Height profile along the white line indicated in (a). (c) Averaged nano-FTIR spectrum obtained from three locations marked in (a).

Custom-made IR s-SNOM system used for monolayer measurements.

To enhance the signal-to-noise ratio for these monolayer measurements, a custom-built IR s-SNOM system (based on neaSNOM, Neaspec GmbH) equipped with a high-repetition-rate pulsed IR laser was employed. As a mid-infrared pulsed light source, we used a tabletop laser system that is essentially identical to the one that has been previously established for its high spectral irradiance and associated sensitivity. The system features a Yb:KGW oscillator (FLINT, Light Conversion) at a center wavelength of 1030 nm, with a pulse duration of ~ 150 fs, a repetition rate of ~76 MHz, and a total output power of ~9 W pumps an optical parametric oscillator (Levante, APE GmbH) to generate signal and idler pulses. The optical parametric oscillator is equipped with an active feedback mechanism to retain a stable output power and spectrum throughout the measurement. Difference frequency generation is conducted between the signal and idler pulses (Harmonixx DFG, APE GmbH) to generate >30 mW of output at a center frequency of ~1660 cm^{-1} and a spectral full-width-at-half-maximum of ~150 cm^{-1}. The resulting mid-infrared pulses are attenuated to <1 mW and used for nano-FTIR measurements. A commercial nano-FTIR spectrometer (neaSNOM, neaspec GmbH), is employed, comprising an atomic force microscope (AFM), an asymmetric Michelson interferometer with an integrated stage, and a HgCdTe detector. The output from the HgCdTe detector undergoes filtering by a high-pass filter (EF505, Thorlabs), amplification (DHPVA-201, FEMTO Messtechnik GmbH), and detection by an external digital lock-in amplifier (HF2LI, Zurich Instruments). The AFM is operated in tapping mode. The lock-in amplifier is referenced by the AFM signal (ω_t), and the signal is demodulated by the harmonics of the tip tapping frequency ($n\omega_t$).

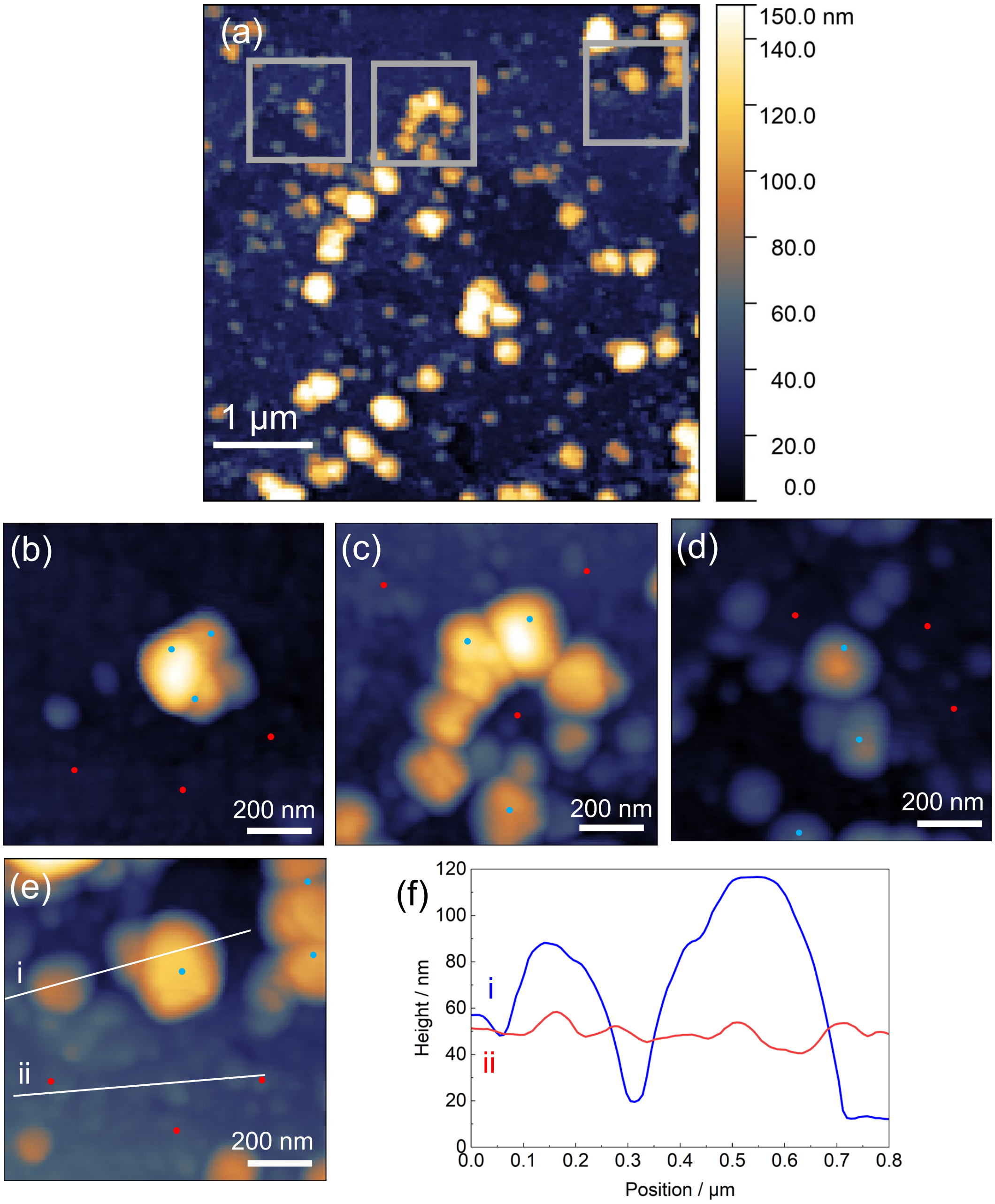

Figure S7. (a) Large-scale AFM image of 10-layer NAFS-1 prepared without water rinsing. (b–e) Enlarged AFM images, where (b) was captured in a region outside of the area shown in (a), and (c–e) correspond to the areas indicated by squares in (a). Red and blue spots indicate the locations where nano-FTIR spectra were recorded; the colors of the spots correspond to the spectra shown in Figure 4. Image (e) is identical to the one used in Figure 4(a). (f) Height profile along the lines indicated in (e).

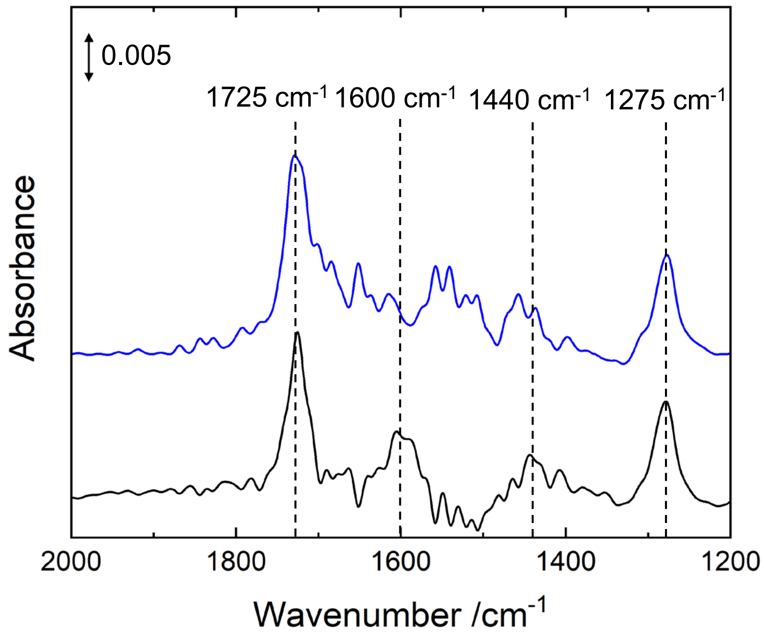

Figure S8. Macroscopic transmission-mode FTIR spectra of NAFS-1 (blue) and NAFS-1 prepared without water rinsing (black).

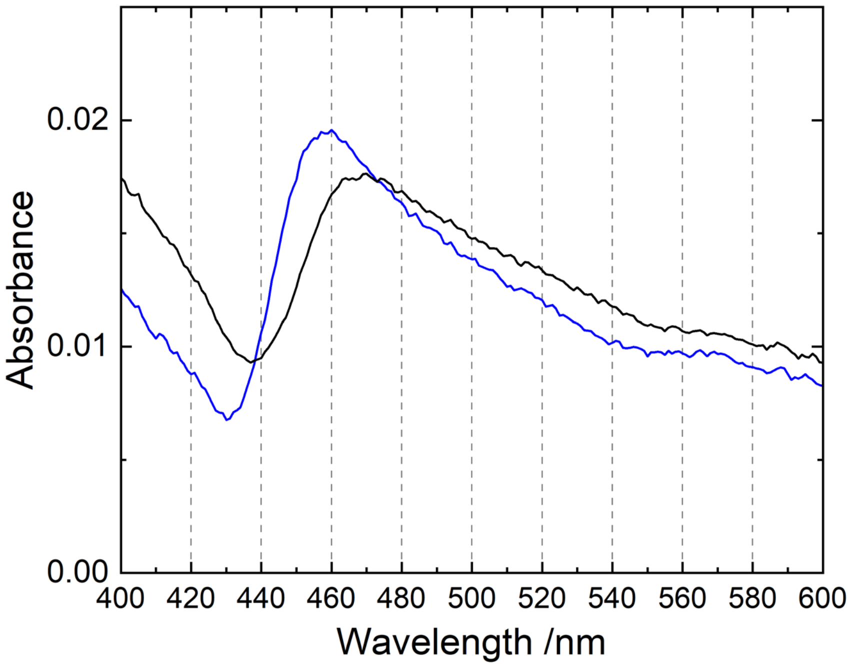

Figure S9. UV-vis absorption spectra of NAFS-1 (blue) and NAFS-1 prepared without water rinsing (black).
